# Postesophagectomy Diaphragmatic Prolapse after Robot-Assisted Minimally Invasive Esophagectomy (RAMIE)

**DOI:** 10.3390/jcm12186046

**Published:** 2023-09-19

**Authors:** Stefanie Brunner, Dolores T. Müller, Jennifer A. Eckhoff, Valentin Lange, Seung-Hun Chon, Thomas Schmidt, Wolfgang Schröder, Christiane J. Bruns, Hans F. Fuchs

**Affiliations:** 1 Department of General, Visceral, Cancer and Transplantat Surgery, University Hospital of Cologne, 50937 Cologne, Germany; 2 Faculty of Medicine, University of Cologne, 50923 Cologne, Germany

**Keywords:** esophagectomy, IL-RAMIE, esophageal cancer, postesophagectomy diaphragmatic prolapse

## Abstract

Background: Postesophagectomy diaphragmatic prolapse (PDP) is a major complication after esophagectomy with significant mortality and morbidity. However, in the current literature, treatment and outcomes are not evaluated for patients undergoing an Ivor Lewis Robot-assisted minimally invasive esophagectomy (IL-RAMIE). The aim of this study is to evaluate the incidence of PDP after IL-RAMIE. Moreover, the study aims to determine whether using a minimally invasive approach in the management of PDP after an IL-RAMIE procedure is safe and feasible. Materials and Methods: This study includes all patients who received an IL-RAMIE at our high-volume center (>200 esophagectomies/year) between April 2017 and December 2022 and developed PDP. The analysis focuses on time to prolapse, symptoms, treatment, surgical method, and recurrence rates of these patients. Results: A total of 185 patients underwent an IL-RAMIE at our hospital. Eleven patients (5.9%) developed PDP. Patients presented with PDP after a medium time of 241 days with symptoms like reflux, nausea, vomiting, and pain. One-third of these patients did not suffer from any symptoms. In all cases, a CT scan was performed in which the colon transversum always presented as the herniated organ. In one patient, prolapse of the small intestine, pancreas, and greater omentum also occurred. A total of 91% of these patients received a revisional surgery in a minimally invasive manner with a mean hospital stay of 12 days. In four patients, PDP recurred (36%) after 13, 114, 119 and 237 days, respectively. Conclusion: This study shows that a minimally invasive approach in repositioning PDP is a safe and effective option after IL-RAMIE.

## 1. Introduction

Esophageal cancer ranks as the sixth most common cause of cancer-related deaths [1]. Multimodal treatment for locally advanced tumors includes neoadjuvant treatment strategies like CROSS radiochemotherapy (CRT) or perioperative FLOT chemotherapy [2,3]. However, 5-year survival remains poor [4].

Esophagectomy is the cornerstone of multimodal treatment for locally advanced esophageal cancer and has significantly improved the 5-year survival rate [5]. Despite improvements in perioperative management and surgical techniques, esophagectomy is still associated with high postoperative morbidity and mortality [6,7].

A rare but potentially morbid complication is a prolapse of intraabdominal organs into the mediastinum [8]. Postesophagectomy diaphragmatic prolapse (PDP) is a transposition of the intraabdominal organs through the hiatal orifice. PDP rarely occurs in the early postoperative cause. It is usually an incidental finding during follow-up visits. In contrast to paraesophageal hernias, which are associated with gastroesophageal reflux, PDP is not covered by a hernial sac. However, the gastric conduit connects the negative intrathoracic pressure with the positive intraabdominal pressure. Hence, transposition of the intraabdominal organs into the chest occurs.

PDP can be associated with aspiration, pneumonia, or bowel obstruction [9]. It accounts for considerable morbidity due to life-threatening emergencies with incarceration, strangulation, and perforation of the bowel [10]. Hence, symptoms range from asymptomatic patients to patients suffering from an acute abdomen.

The pathophysiology of PDP is still under debate. A lack of intraabdominal adhesions and higher intraabdominal pressure might be possible causes for transposition of the intestinal organs into the mediastinum [11].

In the past decade, the surgical technique for performing an esophagectomy has been adapted to a minimally invasive approach [12]. Several studies were able to demonstrate reductions in several postoperative complications with a clear focus on reduced pulmonary complications [13,14]. The ROBOT Trial was able to show the advantages of robotic-assisted minimally invasive esophagectomy (RAMIE). Postoperative pain was reduced after Ivor Lewis-RAMIE (IL-RAMIE) and postoperative quality of life was significantly enhanced [15].

Several studies have described an increase in PDP after minimally invasive esophagectomy (MIE) in comparison to an open approach [16,17]. A recent review found the rate of PDP after MIE to be 4.5% compared to 1% after open esophagectomy, with a possible reason being the lack of postoperative adhesions [18]. However, the occurrence of PDP after IL-RAMIE has, to our knowledge, not yet been assessed.

We previously published the incidence and treatment algorithm for PDP in our Hybrid IL-esophagectomy cohort (laparoscopic abdominal part, open approach for the thoracic part) [9].

The aim of this retrospective study is to complement these results in our robotic cohort by analyzing our patients with PDP after IL-RAMIE for esophageal cancer at our high-volume center.

## 2. Materials and Methods

### 2.1. Patients and Data Collection

This retrospective study was conducted at the Department of General, Visceral, Cancer and Transplant Surgery at the University Hospital Cologne, a European high-volume center (>200 esophagectomies/year) for tumor entities of the upper gastrointestinal tract. Data were retrieved from our prospectively maintained hospital database, “Orbis” (version 97 08043101; Agfa HealthCare N.V., Mortsel, Belgium). Information such as patients’ age, sex, BMI, tumor characteristics, neoadjuvant and adjuvant treatment, as well as comorbidities was collected.

All patients receiving an IL-RAMIE for esophageal cancer between April 2017 and December 2022 were included in this underlying study. The study cohort was followed up for PDP for at least 6 months postoperatively [19].

The patients’ outcomes after surgery for PDP were measured by the length of hospital stay, in-hospital mortality, recurrent prolapse, postoperative complications, and outcomes [20]. Moreover, the surgical technique applied for reposition of the prolapse was analyzed.

### 2.2. Management of Esophageal Cancer Patients

The management of esophageal cancer patients at our center is performed according to national and international standards [21]. We previously published details of our surgical technique and postoperative care [22]. Routine staging procedures, including esophagogastroscopy with biopsies, endoscopic ultrasound, and contrast-enhanced computed tomography scans (CT) of the chest and abdomen, were performed. Before surgery, the patients also underwent cardiac and pulmonary function testing.

Patients with carcinoma staged cT2 or lower received a primary esophageal resection. Patients with an advanced esophageal carcinoma (cT3/4 or N+) received neoadjuvant chemoradiation with paclitaxel, carboplatin, and radiotherapy with 41.4 Gy (CROSS protocol) or chemotherapy with 5-FU, leucovorin, oxaliplatin, and docetaxel (FLOT protocol).

Four to six weeks after neoadjuvant treatment, an IL-RAMIE including two-field lymphadenectomy was performed.

Follow-up evaluations after surgery were recommended every 3 months in the first year, then every 6 months for 2 years, and yearly thereafter. A follow-up CT scan was routinely performed at 6, 12, 18, and 24 months postoperatively.

### 2.3. Technique Description for IL-RAMIE

**Figure 1:**
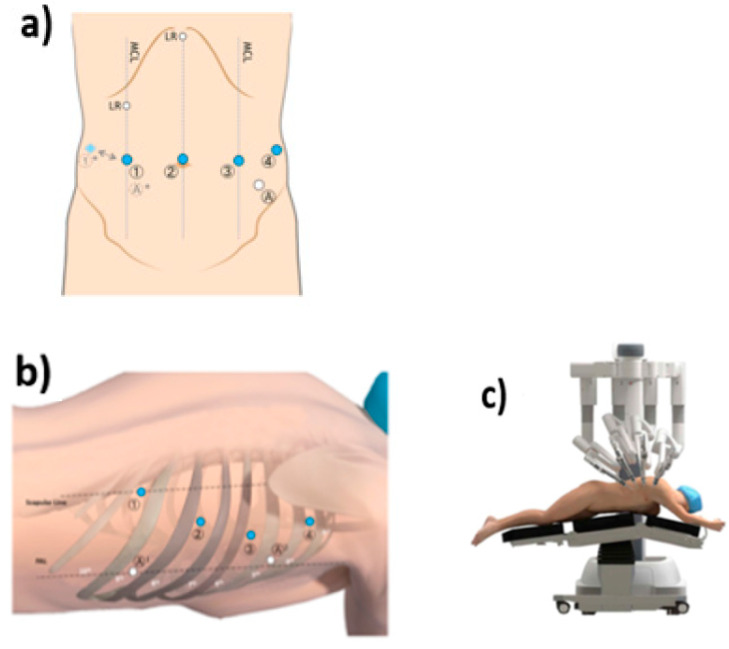
(**a**) Positions of the trocars during the abdominal part of IL-RAMIE. (**b**) Positions of the trocars during the thoracic part of IL-RAMIE (**c**) Positioning of the patient and Da Vinci system during the thoracic part of IL-RAMIE.

The surgery is divided in two parts: the abdominal and thoracic phases. The abdominal phase is performed laparoscopically, while the thoracic part is performed using the Da Vinci Surgical System. The trocar placement is shown in Figure 1. The abdominal phase starts with dissection of the hiatus followed by a D2 lymphadenectomy. Thereafter, the greater curvature is dissected starting at the distal corpus. In the last step of the abdominal phase, the crowfoot region is sought out and a 4 cm wide gastric conduit is created using a linear stapler [22].

Indocyanine green is used to ensure and document sufficient blood supply of the gastroepiploic arcade [23].

For the thoracic phase, the patient is placed in left lateral semiprone position with only the left lung ventilated. The robot is positioned on the right side of the patient. Especially during the thoracic lymphadenectomy and reconstruction, the potential of the robotic technique becomes evident. The dissection is performed starting from the pulmonary ligament upward along the pericardial layer towards the azygos vein using the monopolar cautery hook. Then, the azygos vein is stapled using a tri-stapler (Endo Gia (Covidien), gold, 45 mm). Thereafter, the thoracic duct is dissected and clipped with two clips (Grena Click’V^®^, GRENA Ltd., Nottingham, UK).

In the first step of the reconstruction phase, a monofilament purse string suture is performed. After inserting a 25 or 28 mm stapler head, an anastomosis is created using a circular stapler [22,24].

During an esophagectomy, the esophageal hiatus is commonly widened to facilitate tension-free movement of the gastric conduit. In situations in which the tension on the gastric conduit is too distinct, a relaxing incision in the right hemidiaphragm is performed during the initial operation. However, enlargement of the hiatal opening increases the risk of transposition of the abdominal contents into the pleural cavity or mediastinum. The hiatal opening is not routinely narrowed in our clinic nor is the conduit sutured to the diaphragmatic crura. However, a tailored approach is chosen in our clinic. If the hiatal opening appears too wide, it is loosely closed using non-absorbable sutures.

### 2.4. Management of Patients with Postesophagectomy Diaphragmatic Prolapse

**Figure 2:**
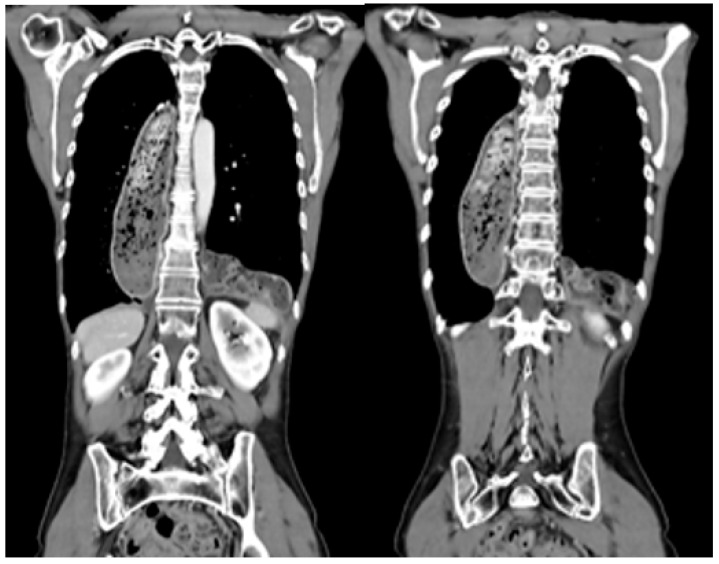
Transposition of colon transversum in one patient after IL RAMIE.

PDP can be suspected based on the patient’s symptoms, such as episodes of abdominal pain, fullness, early satiety, vomiting, and respiratory or cardiac problems. In all patients with PDP-associated symptoms or patients in whom a prolapse was suspected during follow-up examinations, a CT scan was performed (Figure 2). Moreover, routine CT scans were performed in all patients during follow-up. The indication for an elective surgery included an asymptomatic patient and lack of incarcerated organs in the CT scan. Patients presenting with signs of acute incarceration, i.e., acute abdomen, strangulated or perforated conduit, received an emergency surgery.

Generally, revisional surgery was performed using a minimally invasive abdominal approach. A transabdominal laparoscopy was primarily chosen in our patient cohort. No patient underwent surgery using a robotic method or transthoracic approach for the revisional surgery. We placed 4−5 ports. The first step included mobilization of the herniated organs. In most cases, multiple adhesions of the gastric tube occurred. The mobilization was performed carefully in order to avoid lesions of the gastric conduit or the herniated organs. It is important to completely mobilize the crura anterior to the conduit and the posterior part should not be explored if possible, as gastroepiploic vessels typically can be found here. Next, repositioning was performed. The gastric conduit mostly presented itself in a normal position. The herniated organs were then carefully examined to evaluate their perfusion. If the perfusion of the former transpositioned intestines was sufficient, no resection was performed. Only in cases of doubt was a resection performed. Afterwards, the hiatal opening was closed anteriorly using non-absorbable sutures. A mesh as reinforcement was not used in our patient cohort.

### 2.5. Statistical Analysis

The collected data were entered in Excel (Version 14.0.7229.5, Microsoft Corporation, Redmond, WA, USA) and analyzed using SPSS Statistics 24 (IBM Corporation, Armonk, NY, USA). Only a descriptive statistical analysis was performed. All continuous variables were expressed as median and range; all categorical variables were expressed as sum and percentage.

### 2.6. Study Outcome

The primary endpoint was the development of PDP. The secondary endpoints were the time between the esophagectomy and occurrence of PDP, calculated as time from surgery to diagnosis of the prolapse, clinical work-up, diagnostic work-up, content of the herniated organ, its localization, associated complications, treatment, length of hospital stay, in-hospital mortality, and recurrence of PDP.

### 2.7. Approval

The manuscript was submitted to the local ethics committee, which stated that we were exempt from applying for ethical approval as, under German law, no separate ethics application and statement of ethical approval by the local ethics committee is required for performing purely retrospective clinical studies.

## 3. Results

A total of 185 patients underwent an IL-RAMIE for esophageal cancer between April 2017 and December 2022. N = 11 patients developed PDP (5.9%). The cohort was followed up for at least 6 months postoperatively. The mean follow up was 68 months.

The cohort included two female patients (18.18%) and nine male patients (81.81%). The median age at the primary surgery was 56 years (range 43–65 years). The majority of patients were defined as ASA II (54.55%). A relevant amount of the included patients suffered from vascular comorbidities (45.45%) such as peripheral arterial disease. The mean BMI was 25.5 kg/m^2^ (range 18–35 kg/m^2^). Most patients with PDP suffered from adenocarcinoma (90.91%). The tumor was located in the distal esophagus in all cases. None of the patients received a hiatoplasty during the initial surgery. All patients’ characteristics are summarized in Table 1.

In our cohort, five patients were diagnosed with a UICC stadium III and one patient with a UICC stadium IVa. Five patients were postoperatively diagnosed with a lower tumor stadium I or IIb. The distribution of UICC tumor grades was equivalent to that in our total cohort of all patients receiving an esophagectomy in our clinic.

PDP occurred after a medium time of 241 days after IL-RAMIE (range 49–394 days). Symptoms included pain and nausea (36.36%). Two patients complained of dysphagia and reflux (18.8%). One patient suffered from an acute abdomen due to a colon perforation, leading to an emergency operation. Four patients were asymptomatic and PDP was diagnosed during a routine follow-up. All patients received a CT scan for a complete diagnostic work-up. Prolapse occurred most often in the left hemithorax (36.64%). In 27% of the reported PDPs, both the left and right hemithorax were affected. Predominantly, part of the colon (90.9%) was among the transpositioned intestines. In one patient, in addition to the colon, the small intestine, greater omentum, and pancreas were among the herniated organs. No intestinal bowel complication or perforation occurred (Table 2).

An emergency operation was performed in one patient who presented with an acute abdomen. All other patients underwent surgery in an elective surgical setting (91%). In the majority of patients, a minimally invasive surgical technique was chosen (91%). In our patient cohort, a transabdominal laparoscopic approach was performed. In one case, the surgical strategy was converted from laparoscopic to an open approach. The prolapsed colon was strongly fixated in the hiatal region so that the risk of perforation was estimated to be high. The transpositioned intestinal organs were first repositioned in the abdominal cavity. A hiatoplasty was performed in all patients. In four patients, an additional diaphragmatic fixation of the gastric conduit was performed. One-third of the patients received a thoracic drainage. No intestinal bowel resection had to be performed.

The average hospital stay after an operation for PDP was 12 days (range 4–23 days). During the hospital stay, a barium swallow was performed in order to document the sufficient repositioning of the former transpositioned organs and to ensure sufficient esophagogastric emptying. After 3–5 days postoperatively, an oral feeding was conducted. A gradual increase in nutriments was performed. All patients were discharged when the uptake of solid food was possible.

PDP recurred in four patients (36%) after 13, 114, 119 and 237 days, respectively (Table 3).

## 4. Discussion

This retrospective study of our high-volume center provides an overview of the incidence, patient cohort, and operating technique for PDP after IL-RAMIE. With increased survival rates, an increasing number of patients suffering from PDP will potentially need treatment in the future.

PDP has shown to be a late onset yet severe complication following Ivor-Lewis esophagectomy. The risk factors were previously described [25]. Preoperative radiotherapy is stated to be a possible cause for the development of PDP. Especially in the distal esophageal cancers included in this series, 63.64% of the patients received radiotherapy, hence supporting the existing data. It is proposed that radiation weakens the hiatal tissue and hence promotes the development of PDP. Impaired wound healing after chemotherapy could also be a contributory cause [26].

Neoadjuvant therapy is further used for treatment of advanced tumor stages, which leads to a more extensive dissection at the level of the hiatus during the operation. This technical aspect might also contribute to the observed increased risk of PDP in patients after neoadjuvant treatment. Moreover, modern multimodal treatments have resulted in improved long-term survival for patients with esophageal cancer, which might have contributed to the observed increase in PDP incidence.

Furthermore, a BMI of <25 kg/m^2^ is described as a possible cause for the development of PDP [25]. Regarding the patients of our center included in this series, the average BMI was 25.51 kg/m^2^. A higher BMI could be a protective factor for PDP as more fat in the mesentery could decrease the mobility of the small intestine and colon. A high amount of fat along the gastric conduit could minimize the mobility of the gastric conduit and help to fill the hiatal space [27]. The risk factors for PDP need to be investigated in future research in order to develop strategies for its prevention.

The tumor stage itself did not emerge as a risk factor in our cohort. Overall, six patients were diagnosed with an advanced tumor stage of UICC stadium III or higher. A total of 45% of the included patients presented with a lower UICC tumor grade (UICC stadium I or IIb). The distribution of UICC tumor grades was equivalent to that in our total cohort of all patients receiving an esophagectomy in our clinic.

In our patient cohort, all patients received CT scans during follow-up visits and when presenting with symptoms of PDP. In our experience, this diagnostic tool is sufficient for the investigation of PDP.

It is important to consider that, in our investigated cohort, four out of eleven patients were asymptomatic regarding PDP. PDP was diagnosed during a routine follow-up. In our cohort, it was important to conduct to a regular follow-up schedule. When suspecting PDP, it is important to keep in mind that PDP occurs most frequently into the left thorax. Suggested reasons are the anatomic position of the lateral segment of the left lobe of the liver, which possibly prevents a prolapse into the right chest. Moreover, adhesions from the gastric conduit to the liver after gastric pull-up predispose the left side to predominance of PDP.

In our center, we advocate for all patients diagnosed with PDP to undergo surgical revision, even if the patient is asymptomatic. If a patient is asymptomatic concerning PDP, a surgery in an elective setting can be performed. Factors that would contradict the decision to operate include the patient’s wishes, life expectancy, or presence of metastases. PDP has the potential to become a progressive condition that could lead to severe and deadly complications. Additionally, there is a high mortality rate when performing an emergency repositioning of PDP of up to 20%. In contrast, when performing an elective surgery, the mortality rate lowers to 0% [9]. Therefore, it is reasonable to perform prompt treatment of PDP.

In our study, the incidence of PDP after IL-RAMIE was 5.9%. The occurrence of PDP after MIE for esophageal carcinoma was stated to be around 5.8% in the literature, which was in line with our findings [13,28]. Oor et al. demonstrated a higher incidence of PDP after total minimally invasive esophagectomy in comparison with an open approach. The incidence of PDP after minimally invasive esophagectomy was stated to be 4.5% vs. 1% after open esophagectomy [18]. The observed higher incidence of PDP after IL-RAMIE compared to open esophagectomy might be multifactorial. The lack of intestinal adhesions after a minimally invasive gastric mobilization may support the prolapse of intestinal organs [29]. Other suggested factors might be progressive hiatal dilatation due to the increased intraabdominal pressure during robotic- and laparoscopic-assisted esophagectomy [30].

A minimally invasive technique in revisional surgery for PDP requires experience. The treated patients have limited respiratory reserves due to the transpositioned organs in the thoracic cavity. This can lead to sudden desaturation or cardiac arrest during a laparoscopy. Moreover, dissection around the hiatus can compromise the vascular supply of the gastric conduit, which can have severe consequences. Traditionally, laparotomy has been the standard access for treatment of PDP. However, previous studies have shown that a laparoscopic approach can also be successfully performed in an acute setting. Moreover, treating PDP using a minimally invasive approach has proven to be safe and feasible [31,32]. In our cohort study, a minimally invasive surgical technique was chosen in 91% of the operated patients. In one patient, conversion to an open approach was necessary due to a strongly fixated colon in the hiatal region leading to a risk of perforation. We chose to perform a hiatoplasty in all patients. The hiatoplasty was performed using a non-absorbable suture. We did not use a mesh since it might lead to irrevocable adhesions and erode into nearby organs. Additionally, a diaphragmatic fixation of the gastric conduit was performed in selected patients. Minimally invasive repair seems to be safe in experienced centers. The treatment algorithm for PDP was previously published from our center [9].

In the current literature, there are several strategies for preventing PDP after esophagectomy. Messenger et al. suggested a fixation of the gastric conduit to the diaphragm in order to reduce the incidence of PDP [33]. Vallböhmer et al. emphasized closing the hiatus using a non-absorbable suture during an esophagectomy [8]. During the initial esophagectomy, the size of the hiatus should be adjusted to the size of the gastric conduit. If the hiatus presents to be too wide, sutures to narrow the diaphragmatic crura should be placed. However, further studies are necessary to evaluate these strategies.

PDP is a relatively rare complication after esophagectomy, but it can lead to significant problems for the affected patient due to eating disorders and life-threatening incarcerations. The average diagnosis of PDP in this study was 241 days after the esophagectomy. An awareness of PDP and early diagnosis are key to preventing a delayed diagnosis and subsequent complications. In our cohort, we found pain and nausea to be the most commonly reported symptoms (36.36%). In patients with suspected PDP, a CT scan was proven to be the safest diagnostic tool for screening and surveillance; hence, it was performed in all of our patients.

In our cohort, PDP recurred in four patients (36%). In the current literature, the recurrence rate is described to be between 0% and 44.4% [27,34]. In particular, physiologic forces like the pressure gradient between the abdomen and chest contribute to PDP recurrence even after a hiatal repair.

Despite the retrospective character of the study, follow-up data for all patients undergoing an IL-RAMIE between 2017 and 2022 were available and completed according to the standardized protocol. Additionally, we performed surgery in all patients who presented with the diagnosis of PDP using a highly standardized approach. The postoperative complications were precisely documented.

This is, to our knowledge, the first study focusing solely on the occurrence, diagnosis, and treatment of PDP after IL-RAMIE, amplifying the current literature on this topic.

However, there were some limitations due to this study’s retrospective and single-center design. More studies are necessary to compare the occurrence of PDP after open IL-esophagectomy and IL-RAMIE.

## 5. Conclusions

In conclusion, PDP is a rare complication in the late postoperative course after esophagectomy. With increased adoption of minimally invasive esophagectomies, the incidence of PDP may rise. In our cohort, it occurred predominantly in patients with a lower BMI (mean 25.51 kg/m^2^) who received radiochemotherapy prior to an esophagectomy. The organ affected most by transposition was the colon transversum, which prolapsed predominantly in the left thoracic cavity. Elective laparoscopic repositioning of the intestinal organs was safe and feasible at our institution. Strategies such as hiatoplasty can be applied during the initial operation. However, having a high awareness of this rare complication in patients presenting with symptoms of pain or nausea after esophagectomy is essential.

## Figures and Tables

**Table 1:** Baseline characteristics of 11 patients with PDP after IL-RAMIE. AC, adenocarcinoma; SCC, squamous cell carcinoma.

Variable	N
Gender (male)	9 (81.81%)
Age (years)	56 (43.65%)
ASA	
I	3 (27.27%)
II	6 (54.55%)
III	2 (18.18%)
BMI (kg/m^2^)	25.51 (18–35.35)
Preoperative comorbidities	
Cardiac	1 (9.09%)
Pulmonary	2 (18.18%)
Vascular	5 (45.45%)
Tumor histology	
AC	10 (90.91%)
SCC	1 (9.09%)
Tumor localization	
Distal esophagus	11 (100%)
Middle esophagus	0 (0%)
Neoadjuvant treatment	
Radiochemotherapy	7 (63.64%)
Chemotherapy	4 (36.36%)

**Table 2:** Characteristics of patients’ with PDP.

Variable	N
Time to diagnosis (days, range)	241 (49–394)
Symptoms	
Asymptomatic	4 (36.36%)
Pain and nausea	4 (36.36%)
Acute abdomen	1 (9.09%)
Dysphagia and reflux	2 (18.18%)
Localization	
Left hemithorax	7 (36.64%)
Right hemithorax	1 (9.09%)
Bilateral	3 (27.27%)
Prolapsed organs	
Colon	10 (90.91%)
Small intestine, colon, pancreas, greater omentum	1 (9.09%)

**Table 3:** Surgical approach and postoperative outcomes after surgery for PDP.

Variable	N
Surgical indication	
Emergency surgery	1 (9%)
Elective surgery	10 (91%)
Surgical approach	
Open	1 (9%)
Laparoscopic	9 (82%)
Conversion from laparoscopic to open	1 (9%)
Surgical procedure	
Hiatoplasty	11 (100%)
Thoracic drainage	4 (36.4)
Diaphragmatic fixation of prolapsed organ	4 (36.4%)
Length of hospital stay (days)	12 (5–23)
In-hospital mortality	1 (9%)
Recurrent PDP	4 (36%)

## Data Availability

Not applicable.
